# Heme oxygenase 1 improves glucoses metabolism and kidney histological alterations in diabetic rats

**DOI:** 10.1186/1758-5996-5-3

**Published:** 2013-01-16

**Authors:** Ellen ON Ptilovanciv, Gabryelle S Fernandes, Luciana C Teixeira, Luciana A Reis, Edson A Pessoa, Marcia B Convento, Manuel J Simões, Guilherme A Albertoni, Nestor Schor, Fernanda T Borges

**Affiliations:** 1 Nephrology Division, Department of Medicine, Universidade Federal de São Paulo (UNIFESP), São Paulo, Brazil; 2 Morphology Department, Universidade Federal de São Paulo (UNIFESP), São Paulo, Brazil

**Keywords:** Heme oxygenase 1, Nitric oxide synthase, Renal function, Diabetic nephropathy, Glucose metabolism

## Abstract

One important concern in the treatment of diabetes is the maintenance of glycemic levels and the prevention of diabetic nephropathy. Inducible heme oxygenase 1 (HO-1) is a rate-limiting enzyme thought to have antioxidant and cytoprotective roles. The goal of the present study was to analyze the effect of HO-1 induction in chronically hyperglycemic rats. The hyperglycemic rats were divided into two groups: one group, called STZ, was given a single injection of streptozotocin; and the other group was given a single streptozotocin injection as well as daily injections of hemin, an HO-1 inducer, over 60 days (STZ + HEME). A group of normoglycemic, untreated rats was used as the control (CTL).

Body weight, diuresis, serum glucose levels, microalbuminuria, creatinine clearance rate, urea levels, sodium excretion, and lipid peroxidation were analyzed. Histological alterations and immunohistochemistry for HO-1 and inducible nitric oxide synthase (iNOS) were assessed. After 60 days, the STZ group exhibited an increase in blood glucose, diuresis, urea, microalbuminuria, and sodium excretion. There was no weight gain, and there was a decrease in creatinine clearance in comparison to the CTL group. In the STZ + HEME group there was an improvement in the metabolic parameters and kidney function, a decrease in blood glucose, serum urea, and microalbuminuria, and an increase of creatinine clearance, in comparison to the STZ group.

There was glomerulosclerosis, collagen deposition in the STZ rats and increase in iNOS and HO-1 expression. In the STZ + HEME group, the glomerulosclerosis and fibrosis was prevented and there was an increase in the expression of HO-1, but decrease in iNOS expression and lipid peroxidation. In conclusion, our data suggest that chronic induction of HO-1 reduces hyperglycemia, improves glucose metabolism and, at least in part, protects the renal tissue from hyperglycemic injury, possibly through the antioxidant activity of HO-1.

## Introduction

Diabetic nephropathy is one of the most serious complications of diabetes and the most common cause of end-stage renal failure. At present, diabetic kidney disease affects about 15%–25% of type I diabetes patients [1] and 30%–40% of patients with type II diabetes [2]. Diabetic nephropathy is characterized by specific renal morphological and functional alterations.

According to clinical studies, the comorbidities of hypertension, glucose intolerance, and insulin resistance are considered the major contributors to the development of metabolic syndrome and diabetes [3-5].

Reactive oxygen species (ROS) play an important role in diabetes and its complications [6,7]. Experimental studies demonstrated the involvement of ROS in the pathogenesis of diabetes [1] and, more importantly, in the development of diabetic nephropathy [8-10]. Free radicals are capable of damaging cellular molecules, DNA, proteins, and lipids, leading to altered cellular functions. Antioxidants capable of neutralizing free radicals are effective in preventing experimentally induced diabetes in animal models [11,12] as well as in reducing the severity of diabetic complications [13]. ROS are generated by the electron transport system in mitochondrial respiration and increase in conditions associated with enhanced oxidation of substrate such as glucose or free fatty acids. Other ROS-generating agents of relevance to insulin-resistant diabetes include uncoupled endothelial nitric oxide [3,7].

Generally, insulin resistance is accompanied by elevated hyperglycemia, free fatty acids, and adipokines, all of which are factors known to increase oxidative stress [14]. There is a consensus that elevated oxidative/inflammatory stimuli unleash the cascade of events that impairs insulin signaling [14]. As such, insulin resistance may be regarded as a state of increased exposure to ROS [14], and thus, strategies that concomitantly reduce oxidative stress and glucose/insulin intolerance may retard and/or annul the complications observed in diabetes.

Among a panel of potential candidate genes related to oxidative stress, the gene that encodes inducible heme oxygenase (HO-1) has attracted much attention, and HO-1 is considered an “emerging molecule” with potent antioxidant, anti-inflammatory, and anti-proliferative effects [15,16].

In a physiological system, many cytoprotective mechanisms are triggered during tissue insult in an attempt to limit injury. For example, the cellular redox state modulates the expression of stress proteins such as hypoxia-inducible factor and heme oxygenase (HO) during cellular defense [17,18]. HO is a microsomal enzyme with inducible (HO-1) and constitutive (HO-2) isoforms [19]. The HO-catalyzed breakdown of heme yields cytoprotective products including bilirubin, ferritin, and carbon monoxide [19]. HO-1 is strongly induced by oxidative stress and it shows cytoprotective effects by the anti-inflammatory, anti-apoptotic, and anti-proliferative action of its end by-products [19]. Recent studies showed that HO-1 induction is protective in many acute and chronic renal insults [20-24].

The present study observed that the induction of HO-1 with hemin improved glucose metabolism, as seen by a decrease in serum glucose and urea, prevented histological changes observed in diabetic animals, and induced an increase in creatinine clearance. The improvement in glucose metabolism may result, in part, from the antioxidant effect of HO-1.

## Material and methods

### Animal treatment

Male Wistar rats weighing 200–250 g, 10 per group, were divided into three groups: the rats in the first group, the STZ group, were treated with tail injections of streptozotocin [from Sigma (St. Louis, Mo., USA), 60 mg/kg dissolved in sodium citrate buffer, pH 4.5]; the rats in the second group, the STZ + HEME group, were treated with tail injections of streptozotocin as well as daily intraperitoneal injections of hemin [0.1 mg/kg] over 60 days; and the rats in the third group, the control (CTL) group, received tail injection of citrate buffer and no hemin.

The animals were allowed free access to standard rat food and tap water during the experimental protocol. At 0 (baseline), 30, and 60 days after the beginning of experimental protocols, blood samples were collected from the lateral tail vein, and the animals were maintained in metabolic cages over 24 h for urine collection. The animals were killed 60 days after the beginning of the experimental protocol, and both the right and left kidneys were removed for histological analysis. Biochemical parameters in the plasma and urine samples were determined. The experimental protocol was approved by the ethics committee of the Universidade Federal de São Paulo.

### Biochemical analysis

Blood samples were taken from the tails of all the rats and plasma glucose concentrations were determined (Accuchek, Boehringer Mannheim, Indianapolis, Ind., USA). The levels of blood creatinine and blood urea were assayed spectrophotometrically according to standard procedures by using commercially available diagnostic kits (Labtest Diagnostica, Minas Gerais, Brazil). Creatinine was determined by a colorimetric method based on the Jafé reaction [25] and the creatinine clearance was assessed. Urea was determined using a colorimetric assay based on urease activity [26]. Levels of creatinine and urea were expressed in mg/dL. Urine sodium concentrations were determined with a Micronal B462 flame photometer (São Paulo, Brazil). Sodium (ENa) excretions were expressed as mEq/24 h.

The concentrations of albumin in 24-h urine samples were assessed using commercially available enzyme-linked immunosorbent assays (ELISA) (Bethyl laboratories, USA). The optical density of each sample was determined using an Ultra Microplate Reader (EL808; Bio-Tek Instruments, Winooski, VT, USA) and expressed as mg/24 h for urine concentrations.

### Lipid peroxidation

Lipid peroxidation was determined in urine samples by quantifying thiobarbituric acid reactive substances (TBARS). The reactive substances combine with thiobarbituric acid, forming a red compound whose concentration can be assessed spectrophotometrically at an absorbance of 535 nm [27]. Malondialdehyde (MDA) was used as the standard curve, and the results were expressed as nM of MDA/mg creatinine. Urine samples were added to a solution of 0.375% thiobarbituric acid, 15% trichloroacetic acid, and 0.25 N HCl (Sigma-Aldrich, Saint Louis, USA), after which they were continuously agitated while being heated to 95°C for 20 min. Subsequently, the samples were allowed to cool to room temperature. The assessment of the creatinine level in the urine samples was performed as previously described and used to normalize the results of MDA.

### Urinary hydroperoxide

Urinary levels of hydroperoxides were measured by the ferrous oxidation with Xylenol Orange, FOX2 assay for lipid hydroperoxide [28]. Briefly, 100 μl aliquots of urine were transferred into microcentrifuge vials with the addition of 900 μl FOX2 reagent. After incubation at room temperature for a further 30 min, the vials were centrifuged at 12,000 rpm at 25°C for 10 min. Absorbance of the supernatant was then determined at 560 nm. Lipid hydroperoxide content in urine samples was determined according absorption molar coefficient (3 10 [4] M^-1^ cm^-1^). The assessment of the creatinine level in the urine samples was performed as previously described and used to normalize the results of urinary hydroperoxides.

### Nitric oxide determination

Nitric oxide was determined in the 24-h urine samples by the Griess method. Briefly, a mixture of 1% sulfanilamide (in 5% H3PO4) and 0.1% naphthylethylenediamine solution (Sigma, Saint Louis, USA) was added to the samples and the absorbance at 546 nm was measured using a GENESYS 2 spectrophotometer (Spectronic Instruments, Rochester, USA). Nitrite, one of the stable metabolites of NO, was then estimated from a standard curve constructed using NaNO2. The urine volume was used to normalize the quantification.

### Histological analysis

The kidneys were dissected along the non-hilar axis and fixed in 10% phosphate-buffered formalin (Erviegas, São Paulo, Brazil). Kidney sections were fixed with 4% buffered paraformaldehyde and then embedded in paraffin (Erviegas, São Paulo, Brazil). Next, 4-μm thick sections were prepared for hematoxylin and eosin (HE; Erviegas, São Paulo, Brazil) staining, and picro-sirius red staining (PC; Erviegas), and then examined by light microscopy in a blinded manner.

Glomerulosclerosis was semiquantitatively evaluated on HE-stained paraffin sections as follows: grade 0, normal glomerulus; grade 1, beginning mesangial expansion/thickening of the basement membrane and/or irregular lumina of capillaries; grade 2, mild/moderate segmental hyalinosis/sclerosis involving less than 50% of the glomerular tuft; grade 3, diffuse glomerular hyalinosis/sclerosis involving more than 50% of the tuft; and grade 4, diffuse glomerulosclerosis with total tuft obliteration and collapse. Results were reported as the mean ± standard deviation and expressed as arbitrary units (AUs).

### Immunohistochemistry

The kidney slices were deparaffinized and rehydrated. Endogenous peroxidase activity was blocked with 5% H_2_O_2_ in absolute methanol for 10 min at room temperature. To expose the antigens, kidney sections were boiled in a target retrieval solution [1 mmol/L tris(hydroxymethyl)aminomethane (Tris), pH 9.0, with 0.5 mM ethylene glycol tetraacetic acid (EGTA)] for 10 min. Nonspecific binding was prevented by incubating the sections in phosphate buffered saline (PBS) containing 1% bovine serum albumin (BSA), 0.05% saponin, and 0.2% gelatin. Sections were incubated with primary antibodies against HO-1 (1: 200, rabbit anti-human; Enzo Life Sciences, Michigan, USA) and iNOS (1: 200, rabbit anti-rat IgG; ABCAM, MA). Protein expression was measured using a streptavidin peroxidase kit (Dako, California, USA). Sections were stained with diaminobenzidine for HO-1 and iNOS detection and then counterstained with hematoxylin. Signals in negative control sections were absent. Digital photomicrographs were taken through a Nikon Eclipse E600 upright microscope equipped with Plan Apo objectives and connected to a Dell workstation computer through the PixeLINK® Microscope Camera Software (Ottawa, Canada). Eight or nine photomicrographs along the kidney cortex were taken and the light brown staining was quantified (Axionvision microscope program) and averaged for each rat. The values obtained were expressed as pixels per field.

### Statistical analysis

The results are expressed as a mean ± SE. The data were analyzed by one-way analysis of variance (ANOVA) followed by the Tukey tests. Nonparametric data were analyzed by the Dunn tests and p < 0.05 was considered statistically significant.

## Results

Table 1 shows the biochemical parameters observed in the three study groups, (STZ), (STZ + HEME), and (CTL).

**Table 1:** Physiological parameters

**Groups**	**CTL**			**STZ**			**STZ + HEME**		
	**day 0**	**day 30**	**day 60**	**day 60**	**day 30**	**day 60**	**day 60**	**day 30**	**day 60**
**BW, g**	278 ± 14	364 ± 24	436 ± 17	264 ± 10	241 ± 15*	255 ± 20*	247 ± 5	213 ± 18*	226 ± 18*
**Uv, ml**	13 ± 2	14 ± 1	12 ± 1	13.1 ± 1	69 ± 11*	57 ± 18*	16 ± 2	83 ± 5*	86 ± 2*
**BG, mg/dl**	86 ± 4	113 ± 12	109 ± 2	84 ± 4	470 ± 24*	554 ± 29*	80 ± 3	557 ± 27*	338 ± 24*^#^
**U, mg/dl**	45 ± 3	47 ± 4	44 ± 2	48 ± 2	91 ± 11*	88 ± 8*	48 ± 2	70 ± 5*	59 ± 6*^#^
**ClCr, ml/min**	-	-	1.02 ± 0.07	-	-	0.12 ± 0.03*	-	-	0.28 ± 0.02*^#^
**UA, mg/24h**	0.50 ± 0.08	0.33 ± 0.06	0.28 ± 0.04	0.81 ± 0.09	1.67 ± 0.98	3.50 ± 1.04*	0.42 ± 0.05	1.05 ± 0.63	0.85 ± 0.42
**UNaV, mEq/24h**	0.7 ± 0.1	0.8 ± 0.1	0.7 ± 0.1	0.7 ± 0.1	2.8 ± 0.8*	0.9 ± 0.3	1.0 ± 0.1	2.4 ± 0.5*	1.4 ± 0.2*

Data were expressed as the mean ± SEM. The significance level for a null hypothesis was set at 5% (p < 0.05). *BW* (body weight), *Uv* (urinary volume), *BG* (blood glucose), *U* (serum urea), *ClCr* (creatinine clearance), *UA* (urinary albumin) and *UNaV* (urinary sodium excretion) were analyzed in CTL, STZ and STZ+HEME groups. (*) versus CTL and (#) versus STZ.

Long-term diabetes induced by streptozotocin was associated with significant increase in the blood glucose level after 30 (STZ: 470 ± 24 versus CTL: 113 ± 12 mg/dL, p < 0.01) and 60 (STZ: 554 ± 29 versus CTL: 109 ± 2 mg/dL, p < 0.01) days of treatment, and progressive body weight loss after 30 (STZ: 241 ± 15 versus CTL: 364 ± 24 g, p < 0.01) and 60 (STZ: 255 ± 20 versus CTL: 436 ± 17 g, p < 0.01) days of treatment in comparison to CTL. Hemin significantly decreased the blood glucose levels (STZ + HEME: 338 ± 24 mg/dL, p < 0.01), but there was no difference in the body weight (STZ + HEME: 226 ± 18 g, p = 0.36) in STZ + HEME animals after 60 days, when compared to the STZ group.

There was a significant increase in diuresis in STZ and STZ + HEME groups after 30 (STZ: 69 ± 11; STZ + HEME: 83 ± 5 versus CTL: 14 ± 1 mL/24 h, p < 0.01) and 60 (STZ: 57 ± 18; STZ + HEME: 86 ± 2 versus CTL: 12 ± 1 mL/24 h, p < 0.01 and p < 0.05 respectively) days when compared to CTL.

The glomerular filtration was assessed by microalbuminuria, and it was observed that the STZ group had a significant increase in microalbuminuria after 30 (STZ: 1.67 ± 0.98 versus CTL: 0.33 ± 0.06 mg/24 h, p = 0.22) and 60 (STZ: 3.50 ± 1.04 versus CTL: 0.28 ± 0.04 mg/24 h, p < 0.01) days (Table 1). Treatment with hemin significantly inhibited the increase in microalbuminuria when compared to the STZ group (STZ: 3.50 ± 1.04 versus STZ + HEME: 0.85 ± 0.42 mg/24 h, p < 0.01).

Renal function was assessed by creatinine clearance and urea levels. Table 1 shows that there was a significant decrease in creatinine clearance in STZ (0.12 ± 0.03 mL/min, p < 0.01) compared to CTL (1.02 ± 0.07 mL/min) after 60 days of treatment. There was a small but significant increase in creatinine clearance in the STZ + HEME (0.28 ± 0.02 mL/min, p < 0.01) group when compared to the STZ group (p < 0.01).

The plasma urea concentration significantly increased in the STZ group (30 days: 91 ± 11, 60 days: 88 ± 8 mg/dL, p < 0.01) when compared to the CTL group (30 days: 47 ± 4, 60 days: 44 ± 2 mg/dL) (Table 1). In the STZ + HEME group (30 days: 70 ± 5, 60 days: 59 ± 6 mg/dL), this increase was significantly prevented after 60 days (p = 0.03) in comparison to the STZ group (Table 1).

Tubular lesion was assessed by excretion of sodium. There was a significant increase in sodium excretion after 30 days (2.8 ± 0.8 mEq/24 h, p = 0.03) but not 60 (0.9 ± 0.3 mEq/24 h, p = 0.66) days in the STZ group and 30 (2.4 ± 0.5 mEq/24 h, p = 0.02) and 60 (1.4 ± 0.2 mEq/24 h, p = 0.01) days in the STZ + HEME group in comparison to the CTL group after 30 (0.8 ± 0.1 mEq/24 h) and 60 (0.7 ± 0.1 mEq/24 h) days, respectively (p < 0.05), but there was no difference in sodium excretion between the STZ and STZ + HEME groups.

**Figure 1:**
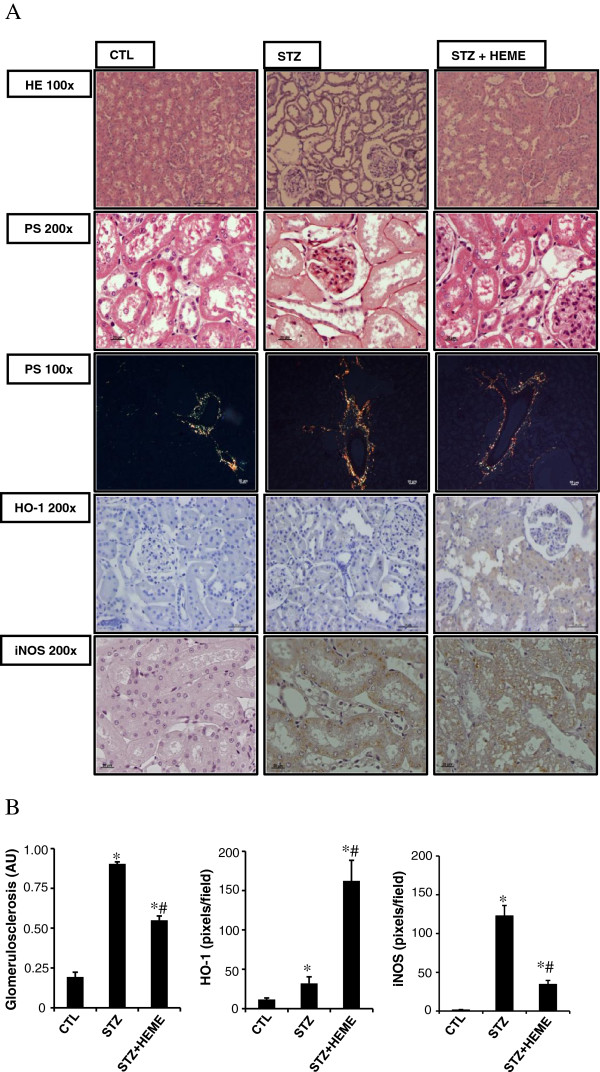
**(A) Light microscopy of kidney sections stained with hematoxylin-eosin at 10x (HE 100), Picro-sirius red 20x (PC 200), 10x (polarized light, PC 100) and immunohistochemistry for HO-1 (200), iNOS (200) in control rats (CTL), rats treated with streptozotocin (STZ), and rats treated with STZ and daily injection of hemin (STZ+HEME). (B)** Quantitative analyses of kidney sections stained for heme-oxygenase-1 (HO-1), induced nitric oxide synthase (iNOS) expressed as pixels/field, stained with HE (glomerulosclerosis) expressed as arbitrary units (AU). Data were expressed as the mean ± SEM. The significance level for a null hypothesis was set at 5% (p < 0.05) (*) significantly different in comparison to CTL group and (#) significantly different in comparison to STZ group.

Figure 1 shows the histological sections of renal tissue staining for HE, and picro-sirius red. When compared with the control group, there was glomerular hypertrophy, collagen deposition in STZ group. There was an improvement in these alterations in the STZ + HEME group, we observed structural collagen deposition. The polarized light showed the immature collagen (green) and intermediate in CTL and SZT + HEME groups, while STZ groups showed mature collagen (red) probably around tubules and vessels. The score of glomerulosclerosis was significantly increased in the STZ group (0.90 ± 0.01 AU) compared with the CTL group (0.19 ± 0.03 AU), whereas hemin treatment (STZ + HEME) reduced the glomerulosclerosis (0.55 ± 0.02 AU) compared with the STZ group.

HO-1 and iNOS staining were examined by immunohistochemistry. There was a small amount of staining for HO-1 (STZ: 32.1 ± 8.4 versus CTL: 11.6 ± 1.9 pixels per field) and a significant increase for iNOS (STZ: 122 ± 13.2 versus CTL: 1.6 ± 0.4 pixels per field) staining in the STZ group in comparison with the CTL group. Treatment with hemin (STZ + HEME) significantly increased the expression of HO-1 (STZ + HEME: 162.2 ± 63.9 pixels per field), as expected, but inhibit the staining for iNOS (34.6 ± 4.8 pixels per field) (Figure 1). We also evaluated the urinary nitric oxide metabolites. We observed an increase in urinary nitric oxide in STZ (43.7 ± 6.8 μM/day) after 60 days of treatment compared to the CTL (6.7 ± 2.4 μM/day) group in 24 h urine volume. In STZ + HEME group (11.3 ± 2.9 μM/day) prevented the increase of urinary nitric oxide.

**Figure 2:**
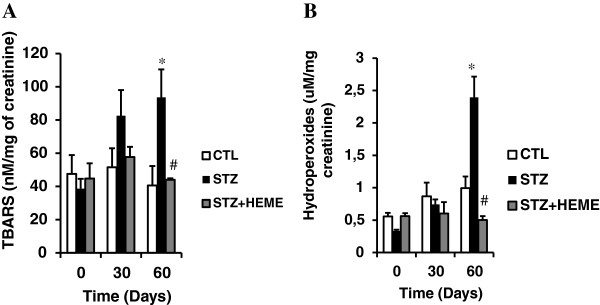
**(A) TBARS levels (nM/mg of creatinine) and (B) Hydroperoxides (μM/mg of creatinine) in the control group (CTL), rats treated with streptozotocin (STZ) and rats treated with streptozotocin + hemin (STZ+HEME) at 0, 30 and 60 days.** Data were expressed as the mean ± SEM. The significance level for a null hypothesis was set at 5% (p < 0.05) (*) significantly different in comparison to CTL group and (#) significantly different in comparison to STZ group.

There was no difference in the lipid peroxidation between the groups at baseline; however there was a significant increase in TBARS in the STZ group (STZ: 93.5 ± 17.0 versus CTL: 47.5 ± 11.0 nM MDA/mg of creatinine) after 60 days (p < 0.05). Additionally, we observed a significant decrease in TBARS in the STZ + HEME group (STZ + HEME: 44.0 ± 0.8 versus STZ: 93.5 ± 17.0 nM MDA/mg of creatinine) compared with the STZ group in the same period (Figure 2). This result was corroborated by the hydroperoxide production. There was an increase in STZ group (STZ: 2.39 ± 0.31 versus CTL: 0.99 ± 0.18 μM/mg of creatinine) after 60 days that was inhibited in the STZ + HEME group (STZ + HEME: 0.50 ± 0.05 μM/mg of creatinine).

## Discussion

One of the major concerns of current diabetes treatment is the maintenance of glycemic levels within a narrow range, reflecting the recommendations of the Diabetes Control and Complications Trial [29]. However, such tight glycemic control may not be feasible, and even patients who diligently regulate their blood glucose levels are still vulnerable to the many complications that characterize diabetes.

There are obvious therapeutic benefits to studying diabetic nephropathy and evaluating novel antidiabetic strategies that are capable of improving the chronic consequences of hyperglycemia.

In the present study, we provide evidence that HO-1 induction could confer protection against metabolic alterations and, at least in part, in kidney histological alterations observed in diabetic animals.

HO-1, the inducible isoform of the HO system, is a rate-limiting enzyme, which converts heme into equimolar amounts of iron, carbon monoxide, and biliverdin. HO-1 is thought to have antioxidant and cytoprotective roles [30]. The products of the HO reaction, biliverdin and carbon monoxide, can be toxic at very high concentrations. However, recent evidence indicates that they are not toxic at physiological concentrations in normal cells, and that they may have important antioxidant, anti-inflammatory, or anti-apoptotic properties [31,32].

The level of HO-1 protein is increased in diabetes. Our work demonstrated that in hyperglycemic rats treated with streptozotocin, the HO-1 expression in the kidneys was increased, and that in similar animals that were treated with hemin, the induction of HO-1 improved the histological alterations observed.

The hyperglycemia induced by streptozotocin promoted diuresis and inhibited weight gain. In our experiment, the treatment with hemin decreased blood glucose and urea, as already observed in other studies. Correa-Costa observed that hemin treatment improved the kidney function and reversed the fibrosis observed in chronic kidney disease induced by ureteral unilateral obstruction [32]. Hemin improved the glucose metabolism in hyperglycemic and spontaneously hypertensive rats [33], and type 2 diabetic subjects showed an increase in HO-1 [34].

In streptozotocin-induced hyperglycemic rats, glomerular alterations such as microalbuminuria, increase in urea levels, and decrease in creatinine clearance, as well as tubular disorders, and increased excretion of sodium were observed. The treatment of hyperglycemic animals with hemin over 60 days inhibited the microalbuminuria, decreased urea levels, and induced a slight increase in creatinine clearance, but did not improve the level of sodium excretion. The effect of hemin on microalbuminuria has also been shown by others [32].

Our histological analysis showed that the renal tissues from diabetic animals treated with hemin were protected from the damage induced by hyperglycemia. Glomerulosclerosis was significantly inhibited and the fibrotic collagen deposition was prevented by hemin treatment.

The protective effects of HO-1 have been demonstrated in vivo; it was found that the induction of HO-1 in mice prevented diabetes-induced kidney injury. This effect resulted from oxidative stress inhibition [35]. Additionally, the interaction between heme oxygenase and nitric oxide (NO) has already been observed in the kidney [36]. The HO-1 induction inhibits NO synthase [37], however fewer works have demonstrated the interaction between these systems in the kidneys of diabetic animals. The present study showed that treatment with hemin also inhibited NO synthase expression and urinary nitric oxide production in 24-h urine, and the inhibition of NO could be involved in the protective effect of hemin.

Histological analysis showed that there was a prevention of tubular injury, fibrosis, induction of HO-1 and inhibition of iNOS and NO in those subjects that had been treated with hemin. This effect may result, in part, from inhibition of lipid peroxidation [33].

In conclusion, our data suggest that chronic induction of HO-1 reduces hyperglycemia, improves glucose metabolism in diabetic animals, and protects the renal tissue from hyperglycemic injury, possibly resulting from antioxidant activity.

## Competing interests

The authors declare that they have no competing interests.

## Authors’ contributios

EONP, GSF, LCT, LAR, EAP and MBC conducted the experiments; EONP and FTB design the experimental protocol; FTB and GAA wrote the manuscript, MJS and NS critically evaluate the results and revised the manuscript. All authors read and approved the final manuscript.
